# Effectiveness of annual influenza campaigns and vaccination in reducing influenza burden in nursing homes of Canton Vaud in Switzerland

**DOI:** 10.1186/s13756-024-01443-z

**Published:** 2024-08-07

**Authors:** Emmanouil Glampedakis, Patricia Cuiña Iglesias, Flaminia Chiesa, Laetitia Qalla-Widmer, May-Kou Ku Moroni, Coralie Riccio, Béatrix Sobgoui, Marie Immaculée Nahimana Tessemo, Alessandro Cassini

**Affiliations:** 1 Cantonal Unit for Infection Control and Prevention, Public Health Service, Lausanne, Switzerland; 2 Cytel Inc, Geneva, Switzerland; 3 La Source School of Nursing, HES-SO University of Applied Sciences and Arts Western Switzerland, Lausanne, Switzerland; 4 Public Health Department, Canton of Vaud, Lausanne, Switzerland; 5 Infectious Diseases Service, Lausanne University Hospital, Lausanne, Switzerland

**Keywords:** Influenza, Nursing home, Long-term care facilities, Vaccination, Mask, Vaccination campaign, Infection prevention and control, IPC

## Abstract

**Background:**

Influenza infections pose significant risks for nursing home (NH) residents. Our aim was to evaluate the impact of the cantonal influenza campaign, and influenza vaccination coverage of residents and healthcare workers (HCWs) on influenza burden in NHs in a context of enhanced infection prevention and control measures (IPC) during the SARS-CoV-2 pandemic.

**Methods:**

We extracted data from epidemic reports provided by our unit to NHs over two consecutive winter seasons (2021-22 and 2022-23) and used linear regression to assess the impact of resident and HCW vaccination coverage, and participation in the campaign, on residents’ cumulative influenza incidence and mortality.

**Results:**

Thirty-six NHs reported 155 influenza cases and 21 deaths during the two winter seasons corresponding to 6.2% of infected residents and a case fatality ratio of 13.5%. Median vaccination coverage was 83% for residents, 25.8% for HCWs, while 87% of NHs participated in the campaign. Resident vaccination was significantly associated with a decrease in odds of death (odds ratio (OR) 0.96, 95% confidence interval (CI): 0.93–0.99). There was no significant effect of HCW vaccination coverage on resident infections and deaths. Campaign participation was associated with decreased odds of infection and death among residents (OR: 0.17, 95% CI: 0.06–0.47 and OR: 0.06, 95% CI: 0.02–0.17 respectively).

**Conclusion:**

Our analysis suggests that in a context of reinforced IPC measures, influenza still represents a significant burden for NH residents. The most effective measures in decreasing resident influenza burden in NHs was participation in the cantonal influenza vaccination campaign and resident vaccination.

**Supplementary Information:**

The online version contains supplementary material available at 10.1186/s13756-024-01443-z.

## Introduction

Infection by influenza virus can have serious consequences for vulnerable populations such as nursing home (NH) residents leading to significant morbidity and mortality [1–3]. Moreover, during winter months, influenza can severely disrupt the functioning of NHs [4] and the quality of life of the residents as isolation measures, suspension of regular activities and often quarantine of whole units are necessary to stop the spread of an outbreak. Additionally, NH healthcare workers (HCW) are at risk of contracting influenza and subsequently transmitting it to residents [5]. As a result, HCW infections can further exacerbate an outbreak and pose additional operational difficulties to the facility due to absenteeism. Thus, it is imperative to maintain continuous vigilance regarding influenza during winter months and promote high vaccination coverage among both residents and staff members, which has been shown to effectively decrease influenza burden in NHs [6–11].

Studies from Switzerland have also confirmed the significant burden of influenza infections for the elderly [12] but data from nursing homes are limited. Similarly, while very few studies have examined the influenza vaccination uptake by the elderly and NH residents [13, 14] less is known about vaccination uptake among staff members of nursing homes.

The Cantonal infection prevention and control unit (HPCi Vaud), part of the Cantonal public health service of Vaud in Switzerland, is responsible for promoting, educating, and setting prevention and control standards in all healthcare facilities, including NHs. As part of these activities, all NHs report influenza cases and outbreaks to the HPCi Vaud unit, which in turn assists NHs in managing them to prevent further spread. HPCi Vaud also elaborates and implements an annual cantonal campaign to raise awareness on influenza prevention and vaccination with a particular emphasis on vaccination of staff while providing NHs with free vaccines.

In a context of enhanced IPC measures in place to mitigate SARS-CoV-2 transmission (mainly universal masking), we aimed to evaluate the impact of participation in the annual cantonal influenza campaign, vaccination of residents and vaccination of HCWs on reducing influenza burden among residents in NHs of the canton Vaud in 2021-22 and 2022-23 winter seasons.

## Methods

### Context

The canton of Vaud is one of the largest cantons of Switzerland (population: ∼830’000 as of 2022) and hosts about 123 NHs (corresponding to ∼ 6000 beds). Over the last decade HPCi Vaud has created and established an extensive IPC network across NHs consisting of IPC-trained and certified link nurses covering 80% of facilities.

IPC link nurses are responsible for ensuring the implementation of IPC best practices and promptly responding to any communicable disease incident within their institutions. Chief nurses and IPC link nurses are also tasked with alerting HPCi Vaud of any new case of communicable disease in their facilities, including influenza cases [15]. Subsequently, HPCi Vaud, discusses and advises on necessary measures with regular follow-ups until the end of the chains of transmission. In the case of influenza, reported incident’s closure is determined after one week from recovery of the last case.

### Temporal extent

The current study focuses on the 2021-22 and 2022-23 winter seasons (from 01 November to 15 April).

### IPC recommendations for NHs

In addition to the standard IPC practices (standard and transmission-based precautions), HPCi Vaud implemented a number of supplementary measures in response to the COVID-19 pandemic for all of the canton’s NHs. These included mandatory face masks for all HCWs regardless of vaccination status upon entering the facility (in 2021-22) or when interacting with residents (in 2022-23), and for symptomatic residents when tolerated. Additionally, PCR testing for SARS-CoV-2 and influenza virus was recommended for all symptomatic residents across the two seasons. For visitors face masks were mandatory upon entering the facility in 2021-22 and in case of respiratory symptoms thereafter.

### Cantonal influenza campaign

HPCi Vaud leads an annual influenza campaign tailored to the NH setting, aiming at raising awareness on prevention and promoting vaccination among HCWs and residents. The campaign is deployed in NHs in October before the onset of the influenza season. This campaign is coordinated by a team of IPC and occupational health physicians and nurses, as well as communication specialists from several French-speaking Swiss cantons. The working group adapts the campaign’s messages and theme yearly.

Campaign materials include A2 and A3 size posters, digital banners and roll-up/totems encouraging vaccination of HCWs, for internal display in NHs. Special flyers are intended for the information and promotion of resident vaccination (see Supplementary material). Additionally, the campaign comprises the provision of free influenza vaccines for HCW on-site vaccination, which is then organized by each NH.

The campaign is financed by cantonal public health funds, and campaign material is provided free of charge to participating institutions. Participation in the campaign is voluntary.

### Data collection

Only NHs reporting influenza incidents (= cases or outbreaks) to HPCi Vaud were included in the study. For each NH, we extracted the cumulative number of resident PCR-confirmed influenza cases and among those the cumulative number of deceased within 30 days, from HPCi Vaud consultation reports. Information regarding the number of beds, residents, HCWs as well as number of vaccinated residents and HCWs and participation of the NH in the cantonal campaign were available via the cantonal HPCi influenza surveillance system. HCW data in this study concerned nurses and nursing assistants. Thus, we were able to calculate the reported seasonal cumulative influenza incidence (number of influenza cases/number of residents at the start of influenza season), reported cumulative mortality (number of deceased among cases/number of residents at the start of influenza season) and the proportion of vaccinated residents and HCWs in each NH reporting cases. Incident-specific parameters, duration of the incident (= time from detection of the first case to withdrawal of droplet precautions for all cases) and attack rate, were also available.

### Statistical analysis

Comparisons involving categorical variables were done using the Pearson’s Chi-squared test whereas for continuous variables the Wilcoxon test was used. We assessed the impact of HCW and resident vaccination and the participation in the annual influenza campaign on resident influenza cumulative incidence and mortality using linear regression. We used a logit transformation for the outcome variables (cumulative incidence and mortality) to account for the fact that these are bounded between 0 and 1. These transformations correspond to the natural logarithm of the odds on infection and death respectively. Because some NHs reported zero deaths a continuity correction was done by adding 0.1 to all deaths and 0.2 to all corresponding resident numbers before the calculation of cumulative mortality and its logit transformation. The resulting regression β coefficients were back transformed by exponentiation and are presented as odds ratios on infection and death with their 95% confidence intervals (CI). A *p* - value < 0.05 was considered statistically significant. All analyses were performed using R Statistical Software (version 4.1.2; R Foundation for Statistical Computing, Vienna, Austria).

### Ethical statement

HPCi Vaud does not collect, store, or report individual-level (resident, HCW) parameters and our data sources comprised aggregated numbers on influenza incidents (cases, deaths) and high scale NH data (numbers of beds, residents, HCWs, vaccinations). Only NH-level data were used, thus our study falls out of the Swiss legislation on human research and an ethics committee approval was not necessary.

## Results

### Participating NHs

In 2021-22, 20 NHs reported 23 influenza incidents corresponding to 90 influenza cases (7.6% of residents) and in 2022-23, 19 NHs reported 20 incidents corresponding to 65 cases (4.8% of residents). Overall (both periods) 36 NHs reported 43 incidents and a total of 155 cases (6.2% of all residents). The detailed characteristics of included NHs together with comparisons between the two study periods are presented in Table 1. Staffing of NHs was similar with 1.5 HCWs per NH bed over the two periods. 79% of NHs had an IPC link nurse and the percentage did not differ significantly between the two study periods (70% vs. 89%, *p* = 0.13).

**Table 1:** Characteristics of included nursing homes and comparisons between the two influenza seasons

Institution characteristics	Season 2021-22	Season 2022-23	Both seasons	*p*- value^1^
NHs included, N	20	19	36^3^	-
Cumulative ^2^ number of NH beds, N	1217	1405	- ^4^	-
Cumulative ^2^ number of residents, N	1188	1363	- ^4^	-
Cumulative ^2^ number of HCWs, N	1995	2081	- ^4^	-
NH beds, median (IQR)	52.5 (36)	76 (48)	61 (41.5)	0.13
NH residents, median (IQR)	49.5 (33.2)	76 (43.5)	61 (37.5)	0.16
NH HCWs, median (IQR)	76.5 (61.5)	101 (71.5)	95 (79.5)	0.27
Percentage of occupied beds, median (IQR)	98.8 (3.5)	100 (2.1)	100 (2.6)	0.22
HCWs per NH-bed, median (IQR)	1.5 (0.5)	1.5 (0.5)	1.5 (0.5)	0.94
NHs with an IPC link nurse, N (%)	14 (70)	17 (89)	31 (79)	0.13

Calculations for both periods have been done over 39 NHs (3 institutions reported cases in both influenza seasons). N: number, %: percentage, NH: nursing home, IQR: interquartile range, HCW: healthcare worker, IPC: infection prevention and control

^1^ Comparisons between the two influenza seasons

^2^ Sum of all included NHs

^3^ 3 NHs announced incidents in both seasons

^4^ Not available

### Incident characteristics

Table 2 summarizes the characteristics of announced incidents. The percentage of infected residents was 7.6% in 2021-22, significantly higher than 4.8% in 2022-23 (*p < 0.01*). The median attack rate was 3.2% (3.6% in the first period and 2.5% in the second period, *p* = 0.44) and the median duration of incidents was 7 days. Two NHs reported 5 deaths in 2021-22 and 4 NHs reported 16 deaths in 2022-23 for a total of 21 fatalities in both periods corresponding to an overall case fatality ratio of 13.5%. Seasonal case fatality ratio was significantly higher in 2022-23 compared to 2021-22 (24.6% vs. 5.6%, *p* < 0.01).

**Table 2:** Characteristics of reported influenza incidents and comparisons between the two influenza seasons

Incident characteristics	Season 2021-22	Season 2022-23	Both seasons	*p*- value^1^
Total influenza incidents announced, N	23	20	43	-
Total number of influenza cases, N	90	65	155	-
Infected residents, %	7.6	4.8	6.2^2^	**< 0.01**
Number of cases per incident, median (IQR)	2 (2)	2 (3)	2 (2.5)	0.83
Attack rate, median (IQR)	3.6% (4.2)	2.5% (4.5)	3.2% (4.5)	0.44
Duration of incident in days, median (IQR)	5 (7)	9 (7.3)	7 (7.5)	0.22
Total number of fatalities, N	5	16	21	-
Case fatality (all incidents), %	5.6	24.6	13.5	**< 0.01**

N: number, %: percentage, IQR: interquartile range

^1^ Comparisons between the two influenza seasons

^2^ Weighted average of the two study periods

### Influenza campaign participation and vaccination

Nineteen NHs participated in the cantonal campaign in 2021-22 (95%) and 15 in 2022-23 season (79%). Although NHs participating in the campaign had generally higher vaccination coverages for both HCWs and residents the differences with those not participating were not significant (median HCW coverage 25.4% vs. 21.7%, *p* = 0.77 and median resident coverage 83.9 vs. 70%, *p* = 0.13). NHs participating in the campaign were not significantly different from those not participating with respect to their number of beds (median 66 vs. 54, *p* = 0.27), number of residents (median 65 vs. 46, *p* = 0.24), number of HCWs (median 99.5 vs. 90, *p* = 0.54) and staffing (median of 1.4 HCWs per NH bed vs. 1.5, *p* = 0.25).

### Other comparisons between nursing homes

When comparing NHs reporting  ≤ 3 influenza cases (27/39, 69%) with those reporting > 3 cases per season (12/39, 31%) no significant differences in NH size, NH staffing or presence of an IPC link nurse were found as shown in Table 3. Although resident and HCW vaccination coverage were higher in NHs reporting up to 3 cases compared to NHs reporting more, the differences were not significant. Participation in the campaign was 96% in the group of NHs reporting  ≤ 3 influenza cases and 67% in those reporting more (*p = 0.01*).

**Table 3:** Comparisons of nursing homes with respect to the number of reported cases and deaths (both study periods)

Characteristic	NHs with ≤ 3 cases (*N* = 27)	NHs with > 3 cases (*N* = 12)	*p*-value		NHs with no deaths (*N* = 33)	NHs with ≥ 1 deaths (*N* = 6)	*p*- value
Beds, Median (IQR)	70 (48)	51 (22)	0.38		61 (50)	57.5 (29)	0.71
Residents, Median (IQR)	68 (43)	47 (23)	0.41		61 (38)	53.5 (32)	0.73
HCWs, Median (IQR)	95 (85)	94 (57)	0.90		95 (86)	95.5 (34)	0.86
HCWs per bed Median (IQR)	1.4 (0.4)	1.6 (0.6)	0.27		1.4 (0.4)	1.4 (0.4)	0.83
NHs with IPC link nurse, N (%)	22 (81)	9 (75)	0.64		26 (79)	5 (83)	0.91
Resident influenza vaccination coverage, Median (IQR)	84% (20)	72% (24)	0.30		84% (20)	65.7% (16)	**0.04**
HCWs’ influenza vaccination coverage, Median (IQR)	27.4% (28.5)	23.8% (14.3)	0.83		25.8% (23)	21.7% (12)	0.72
NHs participating in campaign, N (%)	26 (96)	8 (67)	**0.01**		32 (97)	2 (33)	**< 0.01**

NH: Nursing home, N: number, IQR: interquartile range, HCW: healthcare worker, IPC: infection prevention and control

The comparison of NHs reporting no deaths (33/39, 85%) and those reporting at least one death (6/39, 15%), shown in Table 3, revealed no differences in NH size, NH staffing, and presence of IPC link nurse. Resident vaccination coverage was significantly higher in NHs reporting zero deaths (median coverage 84% vs. 65.7%, *p* = 0.04) but HCW vaccination coverage was not different in the two groups (median coverage 25.8% vs. 21.7%, *p* = 0.72). A significant difference was found between NHs not reporting and those reporting deaths in terms of campaign participation (97% vs. 33%, *p* < 0.01).

### Predictors and outcomes of interest

Vaccination coverage of HCWs (median coverage 25.8%, range 2.2–68.2%) and residents (median coverage 83%, range 52.2–100%) did not differ significantly over the two study periods (*p = 0.43* and *p* = 0.88 respectively). Similarly, although participation in the campaign dropped from 95% in the first period to 79% in the second period, the decrease was not significant (*p = 0.13*). The median cumulative incidence of resident influenza was 0.04 without significant differences between the two winter seasons (*p = 0.18*). Similarly, cumulative resident mortality did not show a significant difference over the two periods (median 0% in both periods, *p* = 0.41) but the overall cumulative mortality was significantly higher in 2022-23 period (0.42 vs. 1.17%, *p* = 0.04). A detailed overview of the distribution of predictors and outcomes of interest is provided in Table 4.

**Table 4:** Distribution of predictors and outcomes of interest and comparisons between the two influenza seasons

	Season 2021-22	Season 2022-23	Both seasons	*p*- value ^1^
**Predictors**				
NHs participating in campaign, N (%)	19 (95%)	15 (79%)	34 (87%)	0.13
Resident influenza vaccination coverage, Median (IQR)	84% (21)	82% (22)	83% (21.8)	0.88
HCWs influenza vaccination coverage, Median (IQR)	29% (27)	18% (18)	25.8% (20.9)	0.43
**Outcomes**				
Cumulative influenza incidence, Median (IQR)	4% (5)	3% (7)	4% (6)	0.18
Cumulative mortality, Median (IQR)	0% (0)	0% (0)	0% (0)	0.41
Cumulative mortality (Overall)	0.42%	1.17%	0.80%^2^	**0.04**

NH: Nursing home, N: number, IQR: interquartile range, HCW: healthcare worker

^1^ Comparisons between the two influenza seasons

^2^ Weighted average of the two study periods

### Regression analysis

The regression models showed a significant association between resident vaccination coverage and odds of mortality. Specifically, an increase of the vaccination coverage of residents by 1% would result in a 4% decrease in their odds of death. There was no significant association between HCW vaccination coverage and the odds of infection or death among residents. Participation in the cantonal campaign was associated with a significant 83% reduction in the odds of infection and 94% in the odds of death (both *p* < 0.01). Tables 5 and 6 summarize the regression model results.

**Table 5:** Odds of resident influenza infection regression model results

Predictor	Odds ratio (95% CI)	*R* ^2^	*p*-value
Resident influenza vaccination coverage			
Season 2021–2022 Season 2022-23 Both seasons	0.98 (0.94–1.03) 0.97 (0.93–1.01) 0.98 (0.95–1.01)	0.03 0.08 0.04	0.48 0.12 0.11
HCW influenza vaccination coverage			
Season 2021–2022 Season 2022-23 Both seasons	1.01 (0.98–1.04) 0.98 (0.94–1.01) 1 (0.98–1.02)	0.03 0.04 0	0.45 0.21 0.98
Campaign participation			
Season 2021–2022 Season 2022-23 Both seasons	0.07 (0.01–0.58) 0.16 (0.05–0.52) 0.17 (0.06–0.47)	0.24 0.35 0.23	**0.02** **< 0.01** **< 0.01**

HCW: healthcare worker

**Table 6:** Odds of resident death regression model results

Predictor	Odds ratio (95% CI)	*R* ^2^	*p*-value
Resident influenza vaccination coverage			
Season 2021–2022 Season 2022-23 Both seasons	0.97 (0.93–1.01) 0.96 (0.90–1.01) 0.96 (0.93–0.99)	0.07 0.09 0.11	0.14 0.12 **0.03**
HCW influenza vaccination coverage			
Season 2021–2022 Season 2022-23 Both seasons	1.01 (0.98–1.04) 0.97 (0.92–1.03) 0.99 (0.97–1.02)	0.02 0.02 0	0.58 0.28 0.64
Campaign participation			
Season 2021–2022 Season 2022-23 Both seasons	0.05 (0.01–0.29) 0.06 (0.01–0.25) 0.06 (0.02–0.17)	0.37 0.47 0.45	**< 0.01** **< 0.01** **< 0.01**

HCW: healthcare worker

## Discussion

The present study, aiming at identifying the impact of different prevention measures in decreasing influenza burden for NH residents showed that resident vaccination and participation in the cantonal influenza campaign demonstrated effectiveness even in a context of reinforced IPC measures.

Few studies have quantified the burden of influenza for NH residents during the SARS-CoV-2 pandemic or the impact of COVID-19 mitigation actions on measures to mitigate influenza infections in NHs. A rapid search in MEDLINE database using the Medical Subject Headings “Influenza” AND “COVID-19” AND (“Aged, 80 and over” OR “Long-Term Care” OR “Nursing Homes”) yielded no reports describing influenza outbreaks in NHs in the post-pandemic era. During the COVID-19 pandemic a sharp decline in influenza cases was published in the scientific literature [16–20]. It has been advocated that the intensification of IPC measures to control the SARS-CoV-2 transmission has been associated with a near disappearance of influenza incidence until late 2021 [21], a feature which has also been observed in Switzerland [22]. Nonetheless, the decrease is expected to subside with the gradual relaxation of SARS-CoV-2 measures. In our study multiple NHs reported influenza cases and outbreaks starting from 2021–22 season and over the two consecutive study periods, affecting approximately 6% of all residents. Although attack rates in our analysis were on the lower bound of what they have been reported in pre-pandemic seasons [1, 2, 23], the case fatality ratio in our analysis (13.5%) approached the upper bounds of those reported in the literature [2]. Case fatality ratio and overall cumulative mortality were especially high in 2022-23 season although the proportion of infected cases was significantly smaller. These discrepancies between the two study periods could be explained by varying viral virulence from season to season [24]. These findings underline the importance of reinstating influenza as a communicable disease priority for NHs each winter season.

Vaccination of residents and HCWs against seasonal influenza has been linked to reductions in influenza-related resident mortality [7–11, 25]. We hypothesized that the impact of vaccination on resident influenza burden might have been attenuated by the reinforced masking policy and other concurrent IPC measures aimed at mitigating SARS-CoV-2 transmission. Overall, we observed a relatively good resident vaccination coverage in both periods (median 83%), however, 41% of included NHs had resident vaccination coverage below the recommended threshold of 75% [26]. We found no significant association between resident vaccination coverage and the odds of resident infection during the study period, consistent with previous studies [5, 23]. Conversely, our study revealed a significant reduction in resident mortality associated with increased resident vaccination coverage which is in line with findings from several other reports [25, 27–29]. Therefore, it remains crucial to maintain efforts to achieve at least 75% vaccination coverage for residents as recommended [26].

HCW vaccination coverage was low in both study periods (median of 25.8%) with substantial variability among institutions (ranging from 2.2 to 68.2%), despite recommendations to HCWs involved in elderly care to get vaccinated against seasonal influenza [30]. Our analysis revealed that in a context of enhanced IPC measures including mandatory masking of HCWs, increasing HCW vaccination did not significantly improve resident outcomes such as the odds of infection and death. Nonetheless, the benefits of HCW vaccination on resident influenza burden have already been established by multiple studies where HCW masking was not present [7–11]. The use of surgical masks has been shown to significantly reduce the spread of respiratory pathogens transmitted by droplets [31, 32] and has been associated with decreasing rates of influenza [21]. Consequently, it is plausible that the use of masks among HCWs has also temporarily “masked” the benefits of HCW vaccination in decreasing the resident influenza burden. With the relaxation of pandemic reinforced IPC measures and given that the elderly exhibit attenuated immunological responses to influenza vaccination [33, 34], HCW vaccination should be more strongly promoted to protect this highly vulnerable population [30].

Influenza campaigns targeting HCWs, which include provision of free vaccines and on-site NH immunization sessions have been demonstrated beneficial in increasing influenza vaccination coverage [35]. In our study, although NHs participating in the campaign had numerically higher percentages of resident and HCW vaccination coverage compared to non-participating institutions, the differences were not statistically significant. A recent study conducted in Belgian NHs showed that a campaign promoting SARS-CoV-2 vaccination decreased resident morbidity and mortality [36]. Our study similarly found a clear advantage for NHs participating in the annual influenza campaigns in both seasons and overall. Reasons could be due to an increased awareness and commitment of participating institutions to appropriate IPC, including rapid surveillance and reporting of cases, hence establishing a swift prevention and control of outbreaks [5, 23]. Moreover, participating NHs displayed posters across their facilities that might have encouraged visitors and/or family members to adhere more strictly to hygiene measures or HCWs to exercise greater caution during care provision and adhere to basic IPC principles such as standard precautions. These possible influences are worth investigating in future research projects.

One of the limitations of the study is its reliance on data from NHs announcing cases to HPCi Vaud and cases occurring in NHs that have not been reported to our unit cannot be entirely ruled out. However, HPCi Vaud has established a robust relationship with most NHs (80%), a lot of which is based on, education, training and prompt reporting and acting on cases of infections. Moreover, in Switzerland healthcare facilities, including NHs, have an obligation to declare outbreaks. Although several systematically collected indicators from NHs of Vaud show similarities with those reported in our study (presence of IPC link nurse: 79%/80%, resident vaccination coverage: 84%/80% in 2021-22 and 82%/78% in 2022-23, HCW vaccination coverage:

29%/27% in 2021-22 and 18%/23% in 2022-23, as reported in this study/from unpublished HPCi Vaud data) suggesting a representative sample, inferences for NHs not represented here need further confirmation by other studies. Another limitation of our study is the absence of data regarding the impact of vaccination and participation in the campaign on influenza transmissions among HCWs, as HPCi Vaud does not systematically collect data for this group. Thus, a potential positive effect of HCW vaccination directly on HCWs as it has been demonstrated in other studies [37, 38] could not be assessed. Finally, it is impossible to draw conclusions regarding the effectiveness of HCW vaccination on the resident influenza burden since masking and other IPC measures might have decreased its impact, as discussed above.

## Conclusions

In conclusion, our study highlights a significant burden from influenza for residents of NHs from the 2021-22 winter season onwards. We showed that even in a context of enforced IPC measures, resident vaccination decreases the odds of mortality among residents, emphasizing the importance of continued promotion of vaccination in this vulnerable population. Finally, influenza campaigns targeting HCWs with sensitization messages through posters and free on-site vaccination appear to confer benefits for residents through reduction of infection transmission and mortality.

### Electronic supplementary material

Below is the link to the electronic supplementary material.

**Supplementary Material 1**: Campaign materials for winter seasons 2021-22 and 2022-23.

## Data Availability

Data (anonymized for nursing homes) and R code used to run the analyses might be obtained upon request to the corresponding author.

## Abbreviations

CI: Confidence interval
COVID-19: Coronavirus disease 2019
HPCi: Cantonal unit for infection prevention and control of Vaud
HCW: Healthcare worker
IPC: Infection prevention and control
IQR: Interquartile range
N: Number
NH: Nursing home
OR: Odds ratio
SARS-CoV-2: Severe acute respiratory syndrome coronavirus 2
