# Supplementary material for: Effectiveness of annual influenza campaigns and vaccination in reducing influenza burden in nursing homes of Canton Vaud in Switzerland
Source: Antimicrob Resist Infect Control. 2024 Aug 7;13:86. doi: 10.1186/s13756-024-01443-z (PMC11304826; doi:10.1186/s13756-024-01443-z)

Cantonal influenza campaign
Season 2021-22

Posters

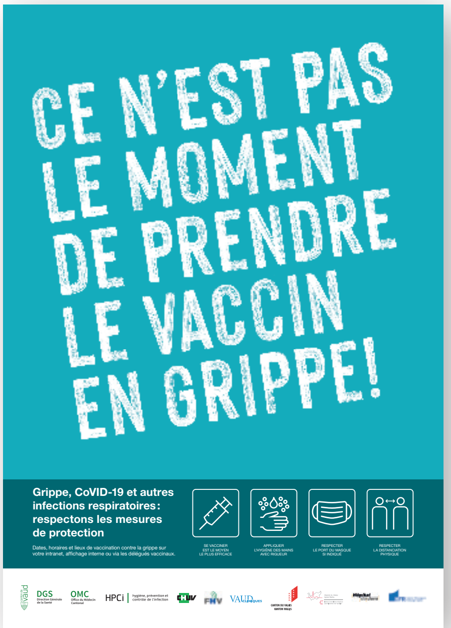

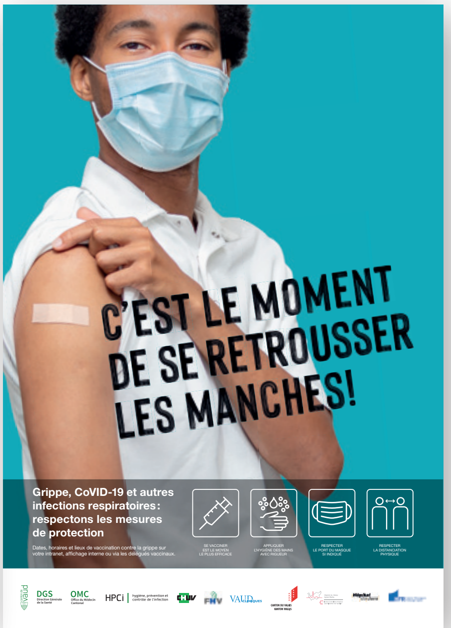

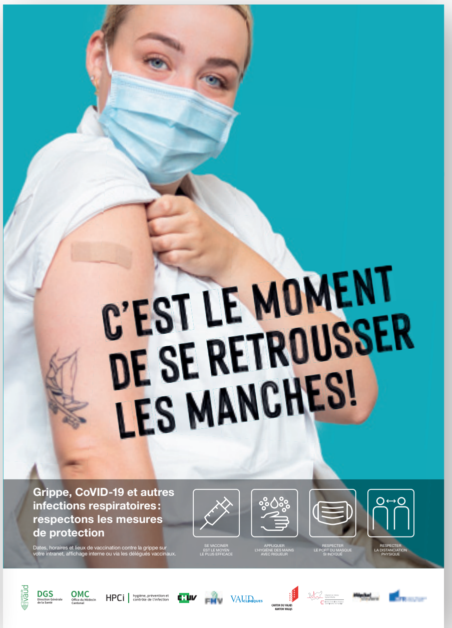

Flyer for residents

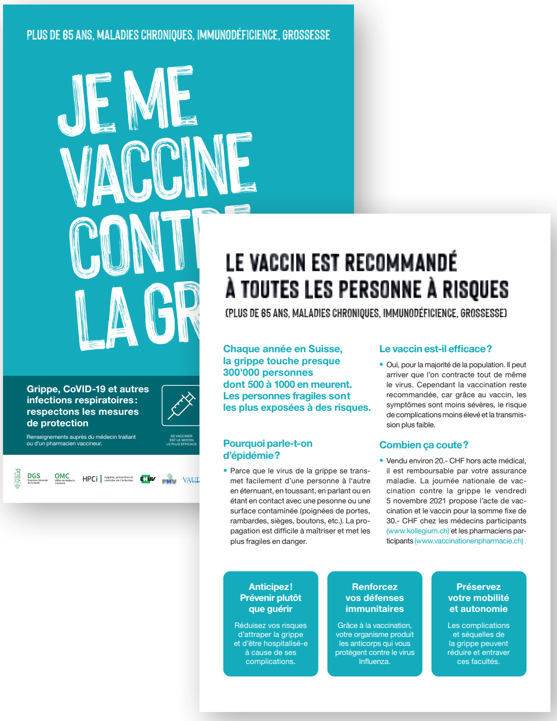

Digital banners

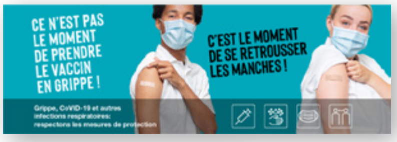

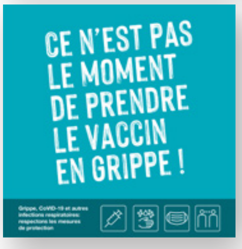

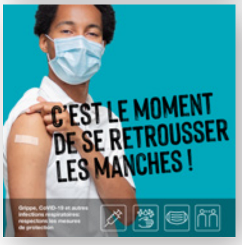

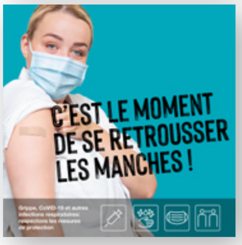

Totems

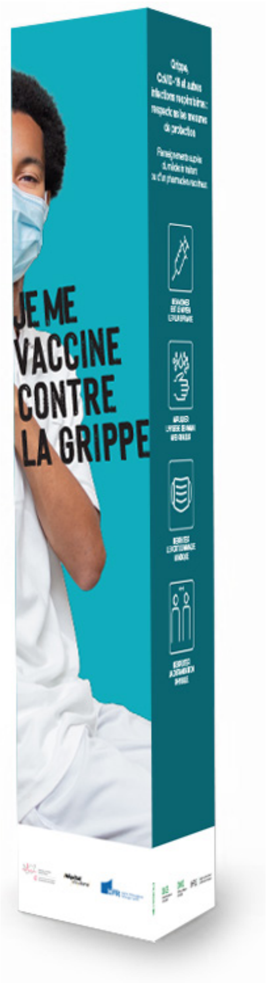

Cantonal influenza campaign
Season 2022-23

Posters

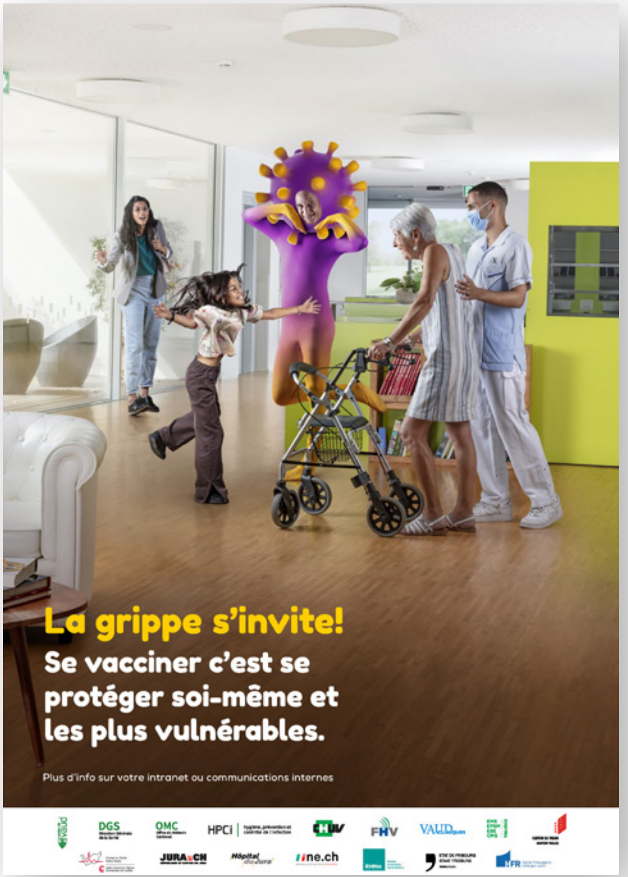

Flyer for residents

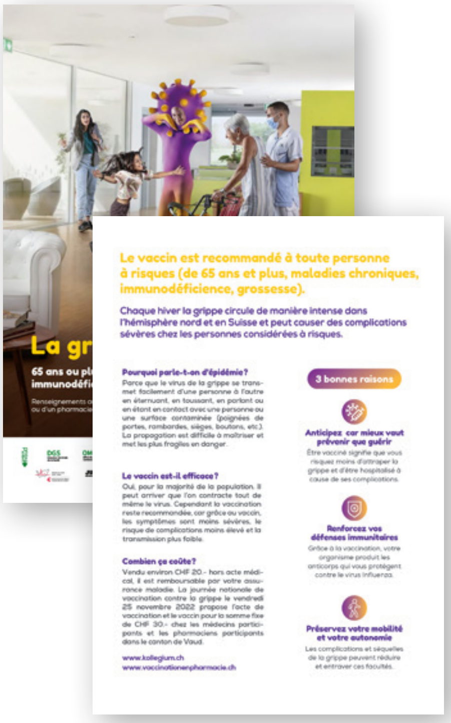

Digital banners

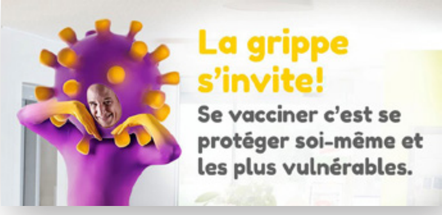

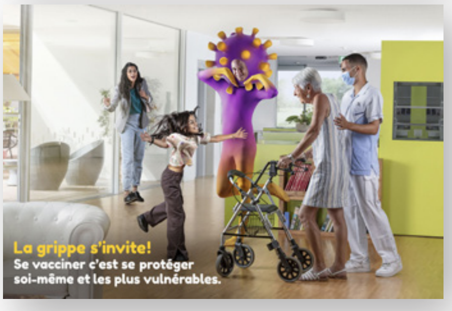

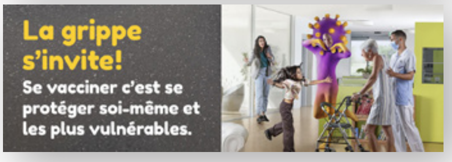

Totems

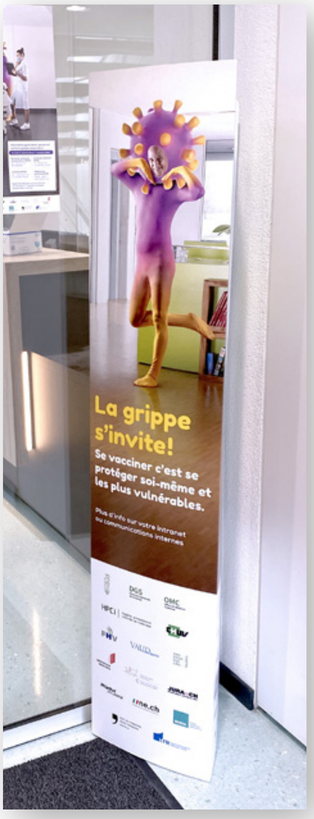

Supplement: Supplementary file 1 — Supplementary Material 1: Campaign materials for winter seasons 2021-22 and 2022-23. [file 13756_2024_1443_MOESM1_ESM.pdf]
